# Women in Medicine

**Published:** 1912-12

**Authors:** 


### Women in Medicine

A trifle less than 65 years ago, in our issue of January, 1848, we were so impressed with the "novel event of a female medical student being in attendance at the Geneva College" that we were "led to state the circumstances * * * as follows:"

"Shortly after the commencement of the lecture session, the faculty received an application for the admission of a lady. The faculty submitted the letter to the class which unanimously adopted resolutions expressing their willingness that the applicant should be received and pledging themselves to treat her with respectful consideration * * * Nothing has transpired as yet to disprove the propriety of the action and, in so far as her presence has had any influence, it has been conducive to a more strict observance of decorum than is usual with medical classes and any embarrassment which may have been felt by all parties has long since disappeared."

Incidentally, there are only four other extant medical journals in the western continent that can speak about "what we did" so far back as the BUFFALO MEDICAL JOURNAL. One of these was suspended for several years during the Civil War and another, so to say, traces its descent through a step parent which it bought.

As there is a prevalent notion that early women medical students encountered opposition and discourtesy, and as this notion is actually correct to a large degree, we are proud to note that western New York manifested a broad-minded spirit both as represented by the student body and faculty of what was at that time a relatively influential medical school and by the JOURNAL. It is by no means improbable that Dr. Corydon L. Ford when he went from the old Geneva school to the University of Michigan took with him not only his personal baggage, dissecting kit and cadaver, but also a tolerance of co-education which had an influence on the latter institution. And, as the University of Michigan was the first educational institution in America to catch the true university spirit, and was the model not only for all other state universities but for the expansion of eastern colleges into universities, it is easy to trace the influence of the courteous reception of this pioneer woman student in western New York, upon the whole course of medical co-education throughout the country.

It is true that at times and in places, prejudice has manifested itself against medical study and practice by women but, as a rule, this prejudice has been of extra-professional origin. Certainly, in recent years, the few complaints of women students and practitioners have mainly been due to a too liberal carrying out of a policy of good fellowship and a forgetfulness of sex by their male associates.

We would define prejudice, not as an academic opinion for or against a given policy but as coercion or annoyance of those who differ from us, or, on the other hand, of undue assistance or applause for those who believe as we do. It is a curious fact that as prejudice against or in favor of medical education of women has disappeared, there has been both an actual and a relative diminution of women medical students. According to the statistics of the A. M. A., since 1904, medical schools for women alone, have been reduced from 3 to 2; the total number of women students in unisexual and co-educational schools has fallen from 1129 to 679 and the yearly graduating class from 244 to 142 . The male graduates have fallen off by almost exactly twenty-five per cent., the female by nearly forty-two per cent. since 1904. Viewed in an other way, in 1905 the women graduates composed 4.3% of the total; in 1912 a little more than 3.3%.

